# PW-360IQA: Perceptually-Weighted Multichannel CNN for Blind 360-Degree Image Quality Assessment

**DOI:** 10.3390/s23094242

**Published:** 2023-04-24

**Authors:** Abderrezzaq Sendjasni, Mohamed-Chaker Larabi

**Affiliations:** CNRS, Université de Poitiers, XLIM, UMR 7252, 86073 Poitiers, France

**Keywords:** 360-degree images, CNNs, scan-path, JND, blind image quality assessment

## Abstract

Image quality assessment of 360-degree images is still in its early stages, especially when it comes to solutions that rely on machine learning. There are many challenges to be addressed related to training strategies and model architecture. In this paper, we propose a perceptually weighted multichannel convolutional neural network (CNN) using a weight-sharing strategy for 360-degree IQA (PW-360IQA). Our approach involves extracting visually important viewports based on several visual scan-path predictions, which are then fed to a multichannel CNN using DenseNet-121 as the backbone. In addition, we account for users’ exploration behavior and human visual system (HVS) properties by using information regarding visual trajectory and distortion probability maps. The inter-observer variability is integrated by leveraging different visual scan-paths to enrich the training data. PW-360IQA is designed to learn the local quality of each viewport and its contribution to the overall quality. We validate our model on two publicly available datasets, CVIQ and OIQA, and demonstrate that it performs robustly. Furthermore, the adopted strategy considerably decreases the complexity when compared to the state-of-the-art, enabling the model to attain comparable, if not better, results while requiring less computational complexity.

## 1. Introduction

Image quality assessment (IQA) is the process of evaluating the visual quality of an image, taking into account various visual factors, human perception, exploration behavior, and aesthetic appeal. The goal of IQA is to determine how well an image can be perceived by human users [1]. IQA is performed objectively using mathematical and computational methods or subjectively by asking human evaluators to judge and rate the visual content. IQA is a challenging and versatile task, as it differs from one application to another and requires a deeper understanding of perception and user behavior to design consistent methodologies. This is especially true for emerging media such as 360-degree (also known as omnidirectional) images that require particular attention to ensure that users can perceive these images accurately and effectively.

**Figure 1:**
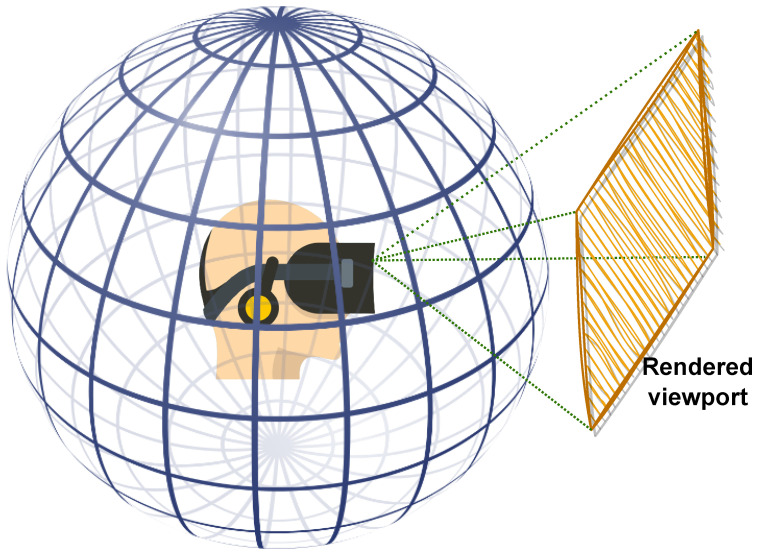
360-degree images viewed using head-mounted devices. Only the current field of view is rendered as a viewport.

360-degree images provide users with the ability to look in any direction of the 360${}^{\circ }$ environment, creating an immersive experience when viewed through head-mounted displays (HMDs) or other specialized devices. As the user gazes in a particular direction, the HMD display renders a portion of the content corresponding to the current field of view (FoV) from the spherical representation, which is effectively a 2D rendered window as illustrated in Figure 1. The next viewport depends on the user’s head movement along the pitch, yaw, and roll axes. This exploration behavior makes processing 360-degree images challenging, particularly for quality assessment, as HVS perception requires appropriate considerations. Additionally, 360-degree images require a sphere-to-plane projection for processing, storage, or transmission, which introduces geometric distortions that must be taken into account when designing IQA methodologies or dealing with 360-degree images, in general [2].

Convolutional neural networks (CNNs) have been successfully used in various fields, including quality assessment, due to their good performance. For 360-degree quality assessment, CNN-based models often adopt the multichannel paradigm, in which multiple CNNs are stacked together to calculate the quality score from selected viewports. However, this architecture can be computationally complex [3] and may not fully consider the non-uniform contribution of each viewport to the overall quality. To address these issues, end-to-end multichannel models that learn the relationship among inputs and the importance of each viewport have been proposed. These models tend to better agree with the visual exploration and quality rating by observers. Moreover, viewport sampling, combined with an appropriate training strategy, may further improve the robustness of the model. A previous study designed a multichannel CNN model for 360-degree images that considers various properties of user behavior and the HVS [4]. However, the model has significant computational complexity.

In contrast to our previous model, this paper introduces a novel approach for 360-degree image quality assessment, called the Perceptually Weighted 360-degree IQA model (PW-360IQA). The proposed model is a multichannel CNN with a weight-sharing strategy, which significantly reduces complexity compared to state-of-the-art models while maintaining higher accuracy. PW-360IQA considers different perceptual characteristics of the HVS, such as visual exploration trajectories and distortion probability maps by utilizing both scan-paths and just-noticeable differences (JNDs). The model first extracts viewports on the spherical content of the 360-degree images based on visual scan-path predictions to reproduce the actual viewed content and avoid severe geometric distortions. Then, motivated by the effectiveness of well known pre-trained CNN models and the observations found in [3], we use DenseNet-121 [5] to extract visual features from the selected viewports and predict their visual quality. To adaptively learn the contribution of each viewport to the overall quality score, a viewport weight estimation is incorporated by fusing learned JND features and visual scan-path attributes such as fixation duration and fixation order. Finally, PW-360IQA estimates a quality score distribution for each input 360-degree image, from which the ultimate score is derived using an adaptive pooling strategy. The primary contributions of this work are:

- The use of the weight sharing strategy to reduce the complexity of the model and improve the robustness of the multichannel CNN.
- Instead of predicting a single score per 360-degree image, a quality score distribution is predicted by taking advantage of the variability of the visual scan-paths.
- Inspired by the way opinion scores are gathered and processed, we define an agreement-based pooling method to derive the final quality scores.

## 2. Related Work

### 2.1. Traditional Models

As 360-degree content has become more popular, a few 360-degree IQA models have been proposed based on the rich literature on 2D-IQA. In particular, Yu et al. [6] introduced the spherical peak signal-to-noise ratio (S-PSNR) which computes the PSNR on a spherical surface rather than in 2D. The weighted spherical PSNR (WS-PSNR) [7] uses the scaling factor of the projection from a 2D plane to the sphere as a weighting factor for PSNR estimation. Similarly, Chen et al. [8] extended the structural similarity index (SSIM) [9] by computing the luminance, contrast, and structural similarities at each pixel in the spherical domain (S-SSIM). The latter uses the same weights as the WS-PSNR. Zakharchenko et al. proposed to compute PSNR on the Craster parabolic projection (CPP) [10] by re-mapping pixels of both pristine and distorted images from the spherical domain to CPP. In contrast, some works, such as those in [11,12,13], incorporate saliency-based weight allocation in the computation of PSNR. Following the trend of adapting 2D models to the 360-IQA domain, Croci et al. [14] proposed extending traditional 2D-IQA metrics such as PSNR and SSIM to 360-degree patches obtained from the spherical Voronoi diagram of the content. The overall quality score is computed by averaging the patch scores. This framework is expanded in [15] by incorporating visual attention as prior to sample Voronoi patches. In a similar vein, the approach proposed by Sui et al. [16] also employs 2D-IQA models for 360-IQA. Specifically, the approach maps 360-degree images to moving camera videos by extracting sequences of viewports along visual scan-paths, which represent possible visual trajectories and generate the set of viewports. The resulting videos are evaluated using several 2D-IQA models, including PSNR and SSIM, with temporal pooling across the set of viewports.

Most traditional 360-degree IQA methods leverage the rich literature on 2D IQA and focus on measuring signal fidelity, which may not adequately capture the perceived visual quality due to the unique characteristics of omnidirectional/virtual reality perception. Furthermore, these models typically obtain the fidelity degree locally using pixel-wise differences, which may not fully account for global artifacts. Additionally, the computation is performed on the projected content, which can introduce various geometric distortions that further affect the accuracy of the IQA scores.

### 2.2. Learning-Based Models

Deep-learning-based solutions, particularly convolutional neural networks (CNNs), have shown impressive performances in various image processing tasks, including IQA. In the context of 360-degree IQA, CNN-based methods have emerged, and they have shown promising results with the development of efficient training strategies and adaptive architectures. The multichannel paradigm is the primary architecture adopted for 360-degree IQA, and viewport-based training is one of the most commonly used strategies. A multichannel model consists of several CNNs running in parallel, with each CNN processing a different viewport, and the quality scores are obtained by concatenating the outputs of these CNNs. The end-to-end model is trained to predict a single quality score based on several inputs. However, a major drawback of this approach is the number of channels or CNNs, which can significantly increase the computational complexity and may affect the robustness of the model [3]. To mitigate this drawback, some techniques such as weight sharing across channels can be helpful. In contrast to multichannel models, a few attempts have been made to design patch-based CNNs for 360-degree image quality assessment, which can reduce the computational complexity. In the following section, we provide a literature review of works that have used different CNN architectures for 360-degree IQA.

#### 2.2.1. Patch-Based Models

Patch-based solutions for 360-degree IQA are mainly used to address the limited availability of training data. By sampling a large number of patches from 360-degree images, the training data can be artificially augmented. Patch-based models consider each input as a separate and individual content item. This makes the training data rich and somehow sufficient for training CNN models. Based on this idea, Truong et al. [17] used equirectangular projection (ERP) content to evaluate the quality of 360-degree images, where patches of 64×64 are sampled according to a latitude-based strategy. During validation, an equator-biased average pooling of patches’ scores is applied to estimate the overall quality. Miaomiao et al. [18] integrated saliency prediction in the design of a CNN model combining the spatial saliency network (SP-NET) [19] for saliency feature extraction, and ResNet-50 [20] for visual feature extraction. The model is trained using cube-map projection (CMP) faces as patches, and is then fine-tuned directly on ERP images. Yang et al. [21] proposed to use ResNet-34 [20] as a backbone where input patches are enhanced using a wavelet-based enhancement CNN and used as references to compute error maps. All these models used projected content to sample the input patches, not taking into account the geometric distortions generated by such a projection nor the relevance of the patch content. Moreover, labeling each patch individually using mean opinion scores (MOSs) regardless of its importance may not accurately reflect the overall quality of the 360-degree image. This is because the local (patch) perceptual quality may not always be consistent with the global (360-degree image) quality due to the large variability of image content over the sphere and the complex interactions between the content and the distortions [22].

#### 2.2.2. Multichannel Models

In the literature, several multichannel models have been proposed for 360-degree IQA. For instance, Sun et al. [23] developed the MC360IQA model, which uses six pre-trained ResNet-34 [20] models. Each channel corresponds to one of the six faces of the CMP, and a hyper-architecture is employed, where the earliest activations are combined with the latest ones. The outputs are concatenated and regressed to a final quality score. Zhou et al. [24] followed a similar approach by using CMP faces as inputs and proposed to use the Inception-V3 [25] model with shared weights to reduce complexity. However, the authors chose not to update the different weights of the pre-trained model, thereby losing the benefits of weight sharing. Kim et al. [26] proposed a model that uses 32 ResNet-50 to encode visual features of extracted 256×256 patches from the ERP images. The 32 channels are augmented with a multi-layer perceptron (MLP) to incorporate patches’ locations using their spherical positions. However, the resulting model is highly complex. It should be noted that predicting quality based on ERP content is not ideal due to severe geometric distortions. Xu et al. [27] proposed the VGCN model, which exploits the dependencies among viewports by incorporating a graph neural network (GNN) [28]. This approach allows for modeling the relationships between viewports in a more effective way. The VGCN model uses twenty ResNet-18 [20] as channels for the sampled viewports, along with a subnetwork based on the deep bi-linear CNN (DB-CNN) [22] that takes ERP images as input. This makes the model significantly complex. Building on the VGCN architecture, Fu et al. [29] proposed a similar solution that models interactions among viewports using a hyper-GNN [30].

As previously mentioned, the use of a higher number of channels in a multichannel model can significantly increase its complexity. Additionally, the learned features from each channel are concatenated automatically, requiring perceptual guiding to consider the importance of each input with respect to the MOS. Relying solely on the CNN to fuse the different representations may not be sufficient. Therefore, a more effective approach is to assist the model in automatically weighting each input based on its importance and significance with respect to various HVS characteristics. In the following section, we describe a multichannel 360-IQA model designed to accurately estimate the contribution of each input by considering different HVS characteristics.

## 3. Proposed 360-Degree IQA Methodology

In this section, we introduce our perceptually weighted 360-degree IQA model (PW-360IQA). The training data are divided into three categories: (1) visual content of chosen viewports, (2) corresponding JND probability maps, and (3) visual trajectory information.

### 3.1. Data Preparation

It is widely acknowledged that 360-degree images in the projected (plane representation) format are subject to geometric distortions caused by the projection process [3,31]. Moreover, the projected content does not correspond to the actual item viewed by users using HMDs. To overcome these issues, we leverage spherical (or radial) content to generate viewports that correspond to the HMD displayed content.

To improve the selection of important viewports from 360-degree images, a visual scan-path is used. This approach is based on the fact that the HVS focuses on salient details when viewing images, resulting in eye fixations [32]. By predicting possible visual trajectories, a visual scan-path model provides insight into the user behavior. Incorporating this information into the design of image quality assessment (IQA) models can be useful. In this study, the scan-path model proposed by Sun et al. [33] is used to generate ten virtual observers (VOs), each consisting of eight ordered fixation points (FPs). These FPs are used as the center of 256×256 viewports (VPs), which are fed to our model as the first category of input data (i.e., the visual content). The scan-path model also provides information about the order and duration of each FP, denoted as $F_{Or}$ and $F_{D}$, respectively. $F_{Or}$ represents the temporal progress of the visual trajectory, while $F_{D}$ indicates the length of time a VP is likely to be focused on. The longer the duration of fixation, the more influence it may have on the observers’ judgment. This information constitutes the second category of data given to our model.

**Figure 2:**
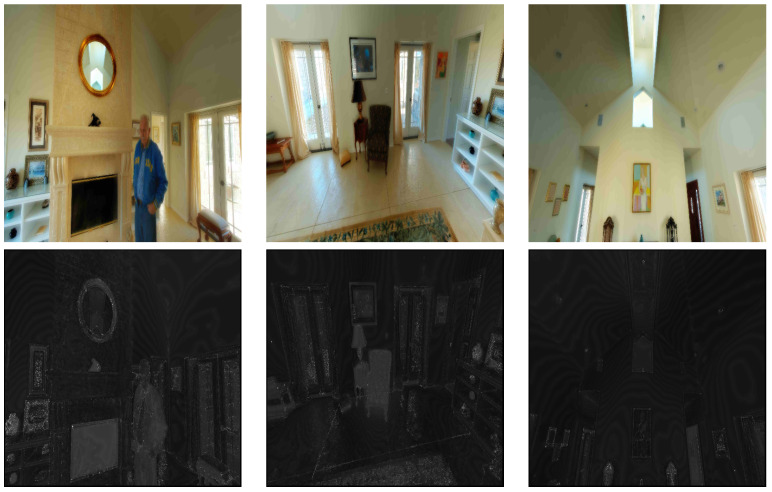
(**Top**) Examples of extracted viewports and (**Bottom**) their corresponding JND probability maps.

To account for the varying sensitivity of different regions in a scene to distortion and the perceptual impact of such distortions on viewports, we use just-noticeable difference (JND) probability maps, denoted as $JND_{map}$, for each VP. These maps provide information about the distribution of degradation over a viewport and across different ones in the same scene. To compute these maps, we utilize the model developed by Wu et al. [34] for 2D images since the viewports are considered as 2D images. Figure 2 shows samples of viewports along with their corresponding JND maps, illustrating the variation in distortion probability values depending on the complexity of each region. Flat regions are more susceptible to visible distortions compared to more complex ones. The JND maps constitute the third category of data given to our model.

**Figure 3:**
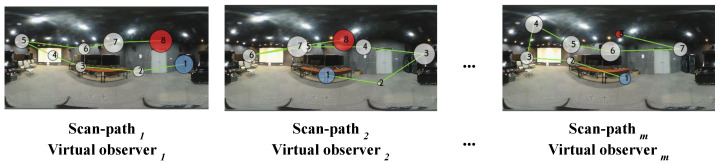
Different scan-paths considered as virtual observers (VOs). Each scan path is composed of eight ordered fixations. The radius of each fixation reflects the fixation duration. Blue represents the first viewed viewport and red corresponds to the last viewport.

As previously mentioned, each 360-degree image *I* is represented as a set of data, consisting of $Vp_{i},JNDmap_{i},\left[F_{Or},F_{D}\right]$, where i∈1…N. The use of different virtual observers (VOs) enables us to account for the diversity of human scan-paths. By considering different VOs, we can mimic exploration behavior and subjective ratings, where each VO explores the same scene differently. Such differences are expressed by (i) the evolution of the visual trajectories and (ii) the fixation duration. Figure 3 depicts examples showing the variability of the generated scan-paths for the same image. As can be seen, each scan-path appears to be distinct compared to the others, and the fixations tend to be located in the equatorial region, mimicking the way users explore 360-degree scenes. Therefore, for *I*, we generate *m* visual scan-paths (SPs) representing the VOs:

(1) $$SPs\left(I\right)=\left\{{\mathrm{VO}}_{0},{\mathrm{VO}}_{1},\ldots ,{\mathrm{VO}}_{m}\right\}.$$

### 3.2. PW-360IQA Model

**Figure 4:**
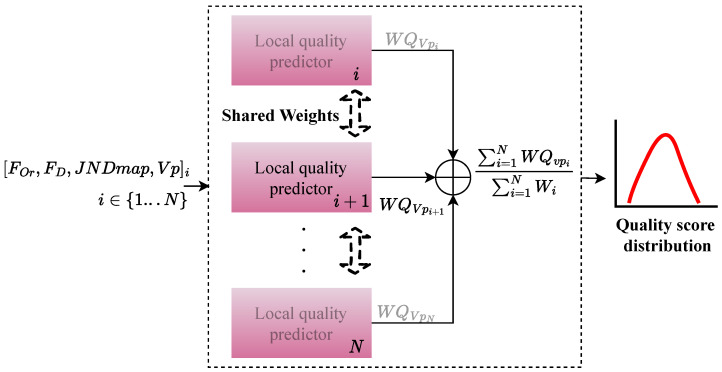
Architecture of the proposed PW-360IQA. ⊕ refers to the summation of $WQ_{Vp_{i}}$.

Figure 4 depicts the architecture of the PW-360IQA model, which adopts a multichannel design with eight channels, each corresponding to a viewport extracted from the 360-degree image. Given a set of viewports $Vp{}_{i}\in R^{+}$ with i∈{0…N} extracted from a 360-degree image, the model takes four inputs including visual content, JND probability map $JND_{map}\in R^{+}$, fixation order and fixation duration $\left[F_{Or},F_{D}\right]\in R^{+}$. These inputs are fed to the local quality predictor (LQP), which consists of eight LQP modules running in parallel, one for each channel. The LQP module fuses the learned representations and outputs a weighted quality score for each $Vp{}_{i}$ denoted as $WQ_{Vp_{i}}$. Finally, the model predicts a weighted arithmetic mean of the local quality scores for each VO, representing a quality score distribution (QSD):

(2) $$QSD_{I}=\left\{f\left(VO_{1}\right),f\left(VO_{2}\right),\ldots ,f\left(VO_{m}\right)\right\},$$

where f(.) represents the model PW-360IQA, and $f(VO_{i})$ is obtained as follows:

(3) $$f\left(VO_{i}\right)=\frac{\sum_{i=1}^{N}WQ_{Vp_{i}}}{\sum_{i=1}^{N}W_{Vp_{i}}},$$

where $WQ_{Vp_{i}}$ is obtained by:

(4) $$WQ_{Vp_{i}}=W_{Vp_{i}}\times Q_{Vp_{i}}.$$

The weight sharing technique is used to reduce the number of parameters to train and to encourage the model to learn the relationships among the input data from the *N* viewports. In weight sharing, the learned weights of the LQP module are updated identically for the eight channels using back-propagation. This means that each LQP is considered as a sub-network, and the weights are computed taking into account the learned relationship among input data from the *N* viewports. By sharing weights across sub-networks, the model requires fewer data and is less likely to over-fit. The architecture of the PW-360IQA model is inspired by the “Siamese Network” [35], where the eight LQPs are considered as mirrored models. This is because each sub-network essentially produces a representation of its input, and since the inputs of each LQP are of the same composition, it appears natural to use the same model to process similar inputs. This allows the LQPs to share the same weights and biases, making it easier to train the model and reduce the number of parameters.

The final quality score of *I* is computed using agreement-based pooling. This method involves discarding scores that are far from the median of the quality score distribution $QSD_{I}$ and only aggregating those within a certain agreement interval. The idea behind this approach is to mimic how subjective ratings are obtained, where only scores that are in agreement with each other are considered for computing the mean opinion score (MOS). Scores that do not agree are detected using the standard deviation σ(.) of $QSD_{I}$. Specifically, a score is rejected only if it falls outside the interval $\left[-\lambda *\sigma \left(QSD_{I}\right),+\lambda *\sigma \left(QSD_{I}\right)\right]$, where $\lambda \in R^{+}$ is a positive real number that determines the appropriate agreement range with respect to the variability of $QSD_{I}$. The resulting quality score distribution with the retained scores is denoted as $QSD_{I}^{\prime }$. Formally, the final score pMOS is obtained as:

(5) $$pMOS_{I}=\frac{1}{|QSD^{\prime }|}\sum_{j\in QSD^{\prime }}QSD_{I_{j}}^{\prime }.$$

#### 3.2.1. Local Quality Predictor

**Figure 5:**
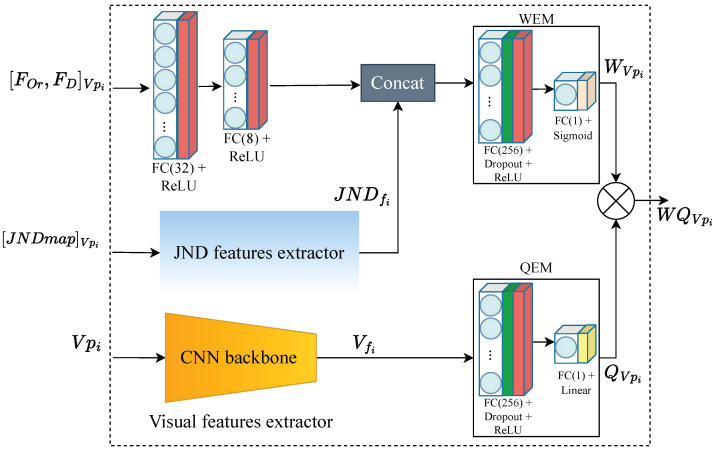
Local quality predictor (LQP) architecture. WEM and QEM stand for the weight and quality estimation modules, respectively, and ⊗ refers to the weighting function of $Q_{Vp_{i}}$.

The proposed PW-360IQA model utilizes the LQP module as its main component, as depicted in Figure 4. The LQP module is composed of three parts, and its architecture is shown in Figure 5. The first part is the visual feature extractor (VFE) where a pre-trained CNN model is used to encode visual features from the input viewports (VPs). In this work, the DenseNet-121 model [5] is selected based on our previous research on CNNs for 360-degree IQA [3]. The study involved a comparison of several pre-trained models across different architectures and paradigms, encompassing various CNN architectures and image representations. Specifically, the study assessed seven models, including ResNet-18/34/50 [20], Vgg-16/19 [36], DenseNet-121 [5], and Inception-V3 [25], in terms of visual feature encoding for 360-degree IQA. The results indicate that DenseNet-121 and ResNet-50 are the top-performing models, with DenseNet-121 achieving superior results while exhibiting less complexity than ResNet-50. In addition, when the same configuration was used, involving viewports and the multichannel architecture, DenseNet-121 outperformed ResNet-50 significantly.

The DenseNet-121, pre-trained on the ImageNet [37] dataset, is a neural network composed of dense blocks, where in each block the layers are densely connected, with L(L+1)/2 direct connections, *L* being the number of layers. Each layer in DenseNet receives additional input from all preceding layers and concatenates them with its own feature maps before feeding them to the subsequent layers. This allows the model to reuse low-level features at later stages. The VFE provides a feature representation thanks to DenseNet-121 feature encoding, from which a feature vector $Vf_{Vp_{i}}\in R^{+}$ is derived using of global average pooling (GAP). The latter goes to a quality estimation module (QEM) composed of fully connected (FC) layers with 256 neurons, followed by rectified linear unit (ReLU) activation function and a dropout for regularization. Another FC layer with a single neuron and a linear activation function is used to deliver the quality score $Q_{Vp_{i}}$.

**Figure 6:**
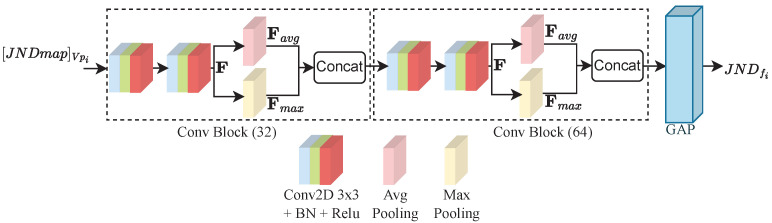
JND feature extractor (JND-FE) architecture. $JND_{f_{i}}\in R^{+}$ represents the feature vector of the JND features. GAP refers to global average pooling.

The second part of the LQP module is the JND features extractor (JND-FE) illustrated in Figure 6. It takes the JND probability map $JNDmap_{Vp_{i}}\in R^{+}$ of $Vp_{i}$ as input and outputs a feature vector $JND_{f_{i}}\in R^{+}$. It comprises two convolutional blocks (Conv Block) with 32 and 64 filters, respectively. Each block is composed of two Conv layers with 3×3 filter size, followed by a batch normalization (BN) layer [38], and then a ReLU activation. BN is used to make the model faster, more stable, and more robust to bad initialization. It is recommended to place BN right after the Conv layers and before ReLU, which helps to produce activations with a stable distribution [38]. All convolutions are used with zero padding to preserve more features and produce an output of the size of the input. After the second activation, a pooling stage is performed where average and max poolings are applied on the refined feature maps *F* obtained from the second activation. The average pooling is applied to account for spatial statistics, whereas max pooling is used to gather distinctive features. The kernels in all pooling layers are of 2×2 size. Two feature maps are obtained at this stage, $F_{avg}$ and $F_{max}$, after applying average and max poolings, respectively. These feature maps are then concatenated to obtain the output features $F^{\prime }$ of each Conv Block. Thereby, $F^{\prime }$ is computed as follows:

(6) $$F^{\prime }=Concat\left(\left[Avg^{2\times 2}\left(F\right);Max^{2\times 2}\left(F\right)\right]\right).$$

Each Conv block outputs features of shape D×H×W, where *H*, *W*, and *D* stand for the height, width, and dimension in terms of the number of channels of the deep features $F^{\prime }$, respectively. *D* is the sum of the number of channels from $F_{avg}$ and $F_{max}$. The produced feature representation by the second Conv block is spatially reduced by the GAP layer to derive the JND feature vector $JND_{f_{i}}$.

The third part is a multi-layer perceptron (MLP) that takes the visual trajectory related information, i.e., $\left[F_{Or},F_{D}\right]$, as input. It is composed of two FC layers of 32 and 8 neurons, respectively. Each layer is followed by a ReLU activation function. The encoded features are then concatenated with $JND_{f_{i}}$ from the JND-FE, before being fed to the weight estimation module (WEM). The latter has the same structure as QEM, except for the final activation used to deliver the weights. Here, a sigmoid activation function is used to estimate the probability of a given VP to be of high or low importance. Therefore, the weight of $V_{P_{i}}$ is obtained as follows:

(7) $$W_{Vp_{i}}=\sigma \left(WEM\left(Concat\left(\left[MLP\left({\left[F_{Or},F_{D}\right]}_{i}\right),JND_{f_{i}}\right]\right)\right)\right).$$

For the end-to-end training of the PW-360IQA model, we used the Huber loss [39], known to be differentiable everywhere and robust to outliers. Hence, the model is then trained to minimize the following loss function:

(8) $$\begin{array}{c} L_{\delta }\left(y,\hat{y}\right)=\left\{\begin{array}{cc} \frac{1}{2}{\left(y-\hat{y}\right)}^{2} & for\;\left|y-\hat{y}\right|\leq \delta ,\\ \delta \left|y-\hat{y}\right|-\frac{1}{2}\delta^{2} & otherwise. \end{array}\right. \end{array}$$

## 4. Experimental Results

In this section, the experimental datasets and evaluation protocols are described. Then, the proposed model is compared with the state-of-the-art quality assessment metrics, followed by an ablation study with a focus on the model design.

### 4.1. Experimental Setup

#### 4.1.1. Datasets

The training and evaluation of the PW-360IQA model are performed on two publicly available 360-IQA datasets, namely OIQA [40] and CVIQ [23]. Details regarding each dataset are given in the following and a summary is provided in Table 1.

OIQA: This includes 320 distorted 360-degree images derived from 16 pristine ones. The used distortions include JPEG compression (JPEG), JPEG 2000 compression (JP2K), Gaussian blur (BLUR), and Gaussian white noise (WN). Each distortion type is applied at five different levels of distortion, resulting in a total of 20 versions for each pristine image.
CVIQ: This includes 16 pristine 360-degree images and their corresponding 528 distorted versions. Only compression-related distortions are used to generate this dataset, namely JPEG with quality factors ranging from 50 to 0, as well as H.264/AVC (AVC) and H.265/HEVC (HEVC) with quantization parameters (QPs) ranging from 30 to 50. Each distortion was applied at eleven levels, resulting in a total of 33 distorted versions for each pristine image.

#### 4.1.2. Implementation Details

PW-360IQA was implemented using TensorFlow and trained on a server equipped with an Intel Xeon Silver 4208 2.1GHz, 192G RAM, and an Nvidia Tesla V100S 32G GPU. The batch size was set to 8 and the Adam optimizer [41] was used with $\beta_{1}=0.9$ and $\beta_{2}=0.999$, following the recommendation in [41] to prevent adverse effects on optimization. The learning rate was initially set to $1\times 10^{-3}$. To provide a complete assessment of the selected datasets, a five-fold cross-validation approach was adopted. Each fold was trained using early stopping techniques by monitoring the validation loss, which ensured that the training stopped when no improvement was observed, thereby avoiding overfitting. During training, the datasets were randomly divided into training, validation, and testing sets. The distorted images derived from the same pristine one were allocated to the same set to avoid an unreliable assessment.

#### 4.1.3. Evaluation Metrics

To evaluate the performance of our model, we used three performance evaluation criteria commonly recommended by the ITU [42]. These included the Pearson linear correlation coefficient (PLCC) for accuracy evaluation, the Spearman rank-order correlation coefficient (SRCC) for monotonicity evaluation, and the root mean squared error (RMSE) for prediction error evaluation. PLCC and SRCC values close to 1 indicate good performance, while values close to 0 indicate poor performance. Lower RMSE values correspond to fewer prediction errors.

### 4.2. Performance Comparison

To demonstrate the effectiveness of our proposed model, we conducted a comparison with state-of-the-art 2D and 360-degree IQA models, including both traditional and learning-based models. The selected models were PSNR, SSIM, BRISQUE [43], S-PSNR [6], WS-PSNR [7], SSP-BOIQA [44], MC360IQA [23], and Zhou et al. [24]. We evaluated the overall and individual distortion performances in terms of PLCC, SRCC, and RMSE, and the results are summarized in Table 2 for CVIQ and Table 3 for OIQA. The performances of the proposed model are reported as the median of five folds. Table 2 also illustrates the complexity comparison in terms of the model capacity, i.e., the number of parameters.

**Figure 7:**
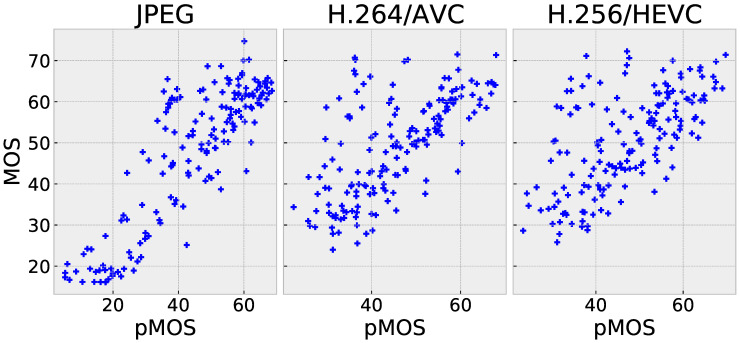
Scatter plots of pMOS vs. MOS of the proposed PW-360IQA model on CVIQ.

Based on the results in Table 2, it can be concluded that the PW-360IQA model achieved the best overall performance on the entire CVIQ dataset, with an accuracy of 0.95. This outperformed both 2D-based and 360-degree IQA models, including the multichannel MC360IQA model and the Zhou et al. model, with improvements of approximately 0.09%/4% in terms of PLCC/SRCC and 5.3%/4.2%, respectively. This is achieved with a significantly low complexity of 7.4M compared to 22M and 29M, respectively. Zhou et al. also applied the weight-sharing technique; however, the model considers only the visual content. For the PW-360IQA, the adopted strategy and architecture appear robust. In terms of accuracy on individual distortions, the proposed model performed competitively with state-of-the-art models, achieving high accuracy on JPEG and AVC with PLCC/SRCC values of 0.971/0.947 and 0.933/0.938, respectively. However, a relatively low accuracy is obtained on HEVC, depicting a difficulty to generalize to some distortions. A scatter plot in Figure 7 shows the distribution of the predictions via the proposed model vs. MOS, where the distribution of predictions for JPEG is more linearly concentrated around the diagonal than for AVC and HEVC. This supports the obtained scores in Table 2.

**Figure 8:**
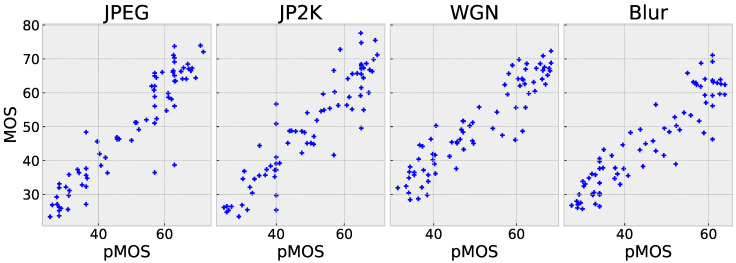
Scatter plots of pMOS vs. MOS of the proposed PW-360IQA model on OIQA.

Table 3 summarizes the performance comparison of different IQA models on the OIQA dataset. The results show that the overall performances on OIQA are relatively low compared to those on CVIQ, which could be attributed to the smaller size of the training dataset in OIQA compared to CVIQ (see Table 1). Despite this, the proposed PW-360IQA achieved robust performances on OIQA, although it scored slightly worse than MC360IQA on the entire dataset. The model’s good performance on individual distortions, such as JPEG and JP2K, where it achieved accuracy of 0.958 and 0.939, respectively, can be supported by the scatter plot in Figure 8, which shows a linearly concentrated distribution around the diagonal. For WGN and BLUR, the points are more spread out, reflecting lower but still competitive performances. Overall, PW-360IQA’s performance across the different distortions in OIQA is quite competitive with state-of-the-art models, and considering the model’s relatively low complexity, it achieves a good trade-off between complexity and performance.

### 4.3. Ablation Study

To thoroughly evaluate the proposed 360-degree IQA methodology, an ablation study is conducted, focusing on both model design and quality score estimation. The aim is to analyze the contribution of each component of the proposed model and assess its effectiveness. A detailed assessment of the results is presented in the following sections.

#### 4.3.1. Model Architecture

PW-360IQA takes various types of inputs as described in Section 3.2. To evaluate the effectiveness of each input and the corresponding module, an analysis of the performances in terms of accuracy, monotonicity, prediction error, and induced complexity is undertaken through an ablation study. Five versions of the PW-360IQA model are benchmarked. First, the quality score is predicted using solely regressed visual content from eight viewports retrieved based on the virtual observer (VO) scan-path. Second, the weight estimation module (WEM) outlined in Section 3.2.1 is included by incorporating visual trajectory information (MLP), distortion detection probability maps (JND), and a combination of MLP and JND. These versions are compared to the final version of PW-360IQA, where weight sharing among local quality predictors (LQPs) is used. The performances of these versions are summarized in Table 4.

Based on the results, it is clear that using only VFE resulted in the worst performances on both CVIQ and OIQA datasets. This highlights the ineffectiveness of relying on a multichannel model to learn the interaction and contribution of each viewport. Therefore, it is necessary to assist the model in completing this task, as demonstrated by the improvement achieved when weight estimation is included. Incorporating fixation order and duration for each viewport led to PLCC (resp. SRCC) values of 0.9473 (resp. 0.9409) on CVIQ, corresponding to a gain of 2.3% (resp. 4.6%). On OIQA, a slight improvement was also observed compared to CVIQ. The nature of the datasets seems to influence the robustness of the model, as JND features resulted in a slight drop in performance on CVIQ but an improvement on OIQA. This can be attributed to the diversity of distortions present in OIQA compared to CVIQ. By combining both visual trajectory information and JND features, similar behavior was observed, supporting the previous observations. The accuracy of the PW-360IQA model is improved on both datasets when weight sharing among LQPs is incorporated. The PLCC/SRCC/RMSE shifted from 0.9372/0.9274/4.9600 to 0.9518/0.9507/4.4200 on CVIQ and from 0.8989/0.8865/6.2000 to 0.9135/0.9155/5.7900 on OIQA. This clearly showcases the efficacy of such an architecture for multichannel models, as the same amount of training data leads to a significant improvement in performance with reduced complexity. The capacity of the model is reduced from the 59.3 M parameter to 7.4 M.

#### 4.3.2. Quality Score Estimation

**Figure 9:**
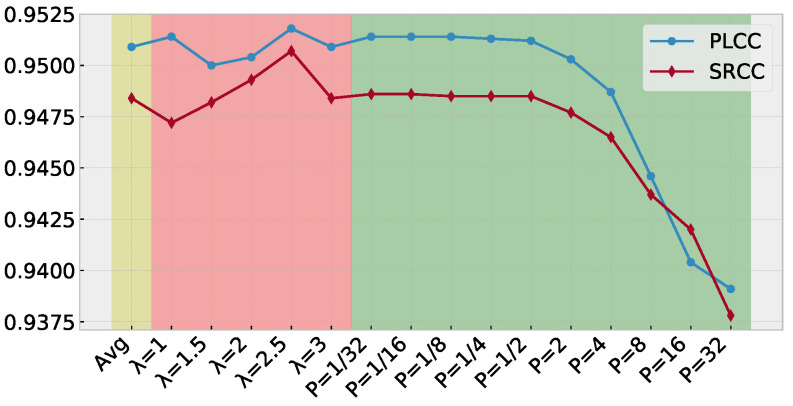
Performances in terms of PLCC and SRCC of the quality score estimation based on the predicted QSD on CVIQ. (yellow) using average pooling, (red) agreement-based pooling, and (green) Minkowski mean.

**Figure 10:**
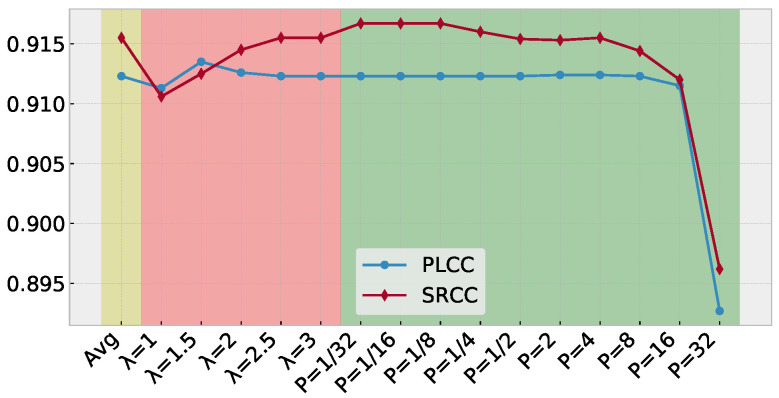
Performances in terms of PLCC and SRCC of the quality score estimation based on the predicted QSD on OIQA. (yellow) using average pooling, (red) agreement based pooling, and (green) Minkowski mean.

The PW-360IQA model generates a QSD for each 360-degree image, as described in Section 3.2. To obtain a representative quality score from QSDs, an agreement-based pooling method is used. This method is compared to a simple average pooling and the Minkowski mean. The performances in terms of PLCC and SRCC on CVIQ and OIQA datasets are provided in Figure 9 and Figure 10, respectively. For the proposed pooling method, the value of λ is set to [1, 1.5, 2, 2.5, 3] to investigate its impact on the performances. In the case of the Minkowski mean, the *P* parameter is varied over the range [1/32, 1/16, 1/8, 1/4, 1/2, 2, 4, 8, 16, 32] to evaluate its influence on the results.

Based on the results obtained on CVIQ, it is obvious that the agreement-based pooling method outperforms the other methods in terms of accuracy. The λ parameter has a significant impact on the correlation performance, with increasing monotonicity and accuracy up to λ=2.5. The best performance is achieved with λ=2.5. The Minkowski mean method exhibits stable PLCC/SRCC, which decreases as *P* increases. These results suggest that the scores within the SQD are highly consistent, indicating that the model predicts similar scores for each VO. Regarding the performances on OIQA, the agreement-based pooling appears less sensitive to the λ value compared to CVIQ. The Minkowski mean shows a similar behavior where the performances are stable, except with P=32. This is probably due to the scores’ range in SQD. The PW-360IQA can predict scores that are quite close, indicating a good fitting of the input data to the MOS.

## 5. Conclusions

In this paper, a novel multichannel convolutional neural network solution with weight sharing for 360-degree image quality assessment is presented. The proposed PW-360IQA model takes advantage of three types of inputs: (a) visually significant viewports extracted from spherical representations of the images, (b) visual trajectory attributes generated using visual scan paths, and (c) distortion probability maps based on just-noticeable difference. The model consists of eight channels, each delivering a weighted quality score for the corresponding viewports. The model is trained to predict a quality score distribution composed of ten scores, from which an overall quality is derived using an agreement-based pooling method. The obtained performances show the efficiency of the proposed model architecture and training strategy. Additionally, the PW-360IQA outperforms state-of-the-art models while showing a significantly reduced complexity compared to other multichannel models. Finally, the weight sharing architecture significantly contributes to the model’s performance improvement.

## Figures and Tables

**Table 1:** Summary of the used 360-degree image databases.

Database	OIQA [40]	CVIQ [23]
Ref images	16	16
Distorted images	320	528
Distortion type (distortion levels)	JPG(5)/WGN(5) JP2K(5)/BLR(5)	JPG(11)/AVC(11) HEVC (11)
Number of observers	20 (M: 15, F: 5)	20 (M: 14, F: 6)
HMD	HTC Vive	HTC Vive

**Table 2:** Performance comparison with state-of-the-art models on the CVIQ dataset. The Best performance is highlighted in **bold** and the second-best is underlined.

		ALL			JPEG			H.264/AVC			H.265/HEVC			Complexity
**Ref.**	**Model**	**PLCC**↑	**SRCC**↑	**RMSE**↓	**PLCC**↑	**SRCC**↑	**RMSE**↓	**PLCC**↑	**SRCC**↑	**RMSE**↓	**PLCC**↑	**SRCC**↑	**RMSE**↓	**# Params**↓
FR	PSNR	0.7320	0.7662	9.0397	0.7342	0.8643	8.5866	0.7572	0.7592	8.0448	0.7169	0.7215	8.3279	
FR	SSIM	0.8857	0.8972	6.2140	0.9334	**0.9749**	3.7986	0.9451	0.9457	4.0165	0.9220	**0.9232**	4.6219	
NR	BRISQUE	0.7448	0.7641	9.0751	0.8489	0.9091	7.1137	0.7193	0.7294	8.4558	0.7151	0.7104	8.4646	
FR	S-PSNR	0.7467	0.7741	8.9066	0.7520	0.8772	8.1974	0.7690	0.7748	7.8743	0.7389	0.7428	8.0515	
FR	WS-PSNR	0.7498	0.7755	8.8816	0.7604	0.8802	8.1019	0.7726	0.7748	7.8143	0.7430	0.7469	7.9974	
NR	SSP-BOIQA	0.8900	0.8561	6.9414	0.9155	0.8533	6.8471	0.8850	0.8611	7.0422	0.8544	0.8410	6.3020	
NR	MC360IQA	0.9506	0.9139	**3.0935**	**0.9746**	0.9316	**2.6388**	0.9461	0.9244	**2.6983**	0.9126	0.8985	**3.2935**	22 M
NR	Zhou et al.	0.9020	0.9112	6.1170	0.9572	0.9611	5.6014	**0.9533**	**0.9495**	3.8730	**0.9291**	0.9141	4.5252	29 M
NR	PW-360IQA	**0.9518**	**0.9507**	4.4200	0.9716	0.9478	5.2499	0.9337	0.9385	4.8085	0.8818	0.8496	5.5504	**7.4 M**

**Table 3:** Performance comparison with state-of-the-art models on the OIQA dataset. The Best performance is highlighted in **bold** and the second-best is underlined.

		All			JPEG			JP2K			WGN			BLUR		
**Ref.**	**Model**	**PLCC**↑	**SRCC**↑	**RMSE**↓	**PLCC**↑	**SRCC**↑	**RMSE**↓	**PLCC**↑	**SRCC**↑	**RMSE**↓	**PLCC**↑	**SRCC**↑	**RMSE**↓	**PLCC**↑	**SRCC**↑	**RMSE**↓
FR	PSNR	0.6910	0.6802	10.388	0.8658	0.8291	7.8570	0.8492	0.8421	7.9357	0.9317	0.9008	4.6392	0.6357	0.6374	10.250
FR	SSIM	0.8892	0.8798	6.5814	0.9409	0.9346	5.3193	0.9336	**0.9357**	5.3829	0.9026	0.8846	5.4965	0.9188	0.9238	5.2404
NR	BRISQUE	0.8424	0.8331	11.261	0.9160	0.9392	8.9920	0.7397	0.6750	15.082	0.9553	0.9372	3.4270	0.8663	0.8508	9.6970
FR	S-PSNR	0.7153	0.7115	10.052	0.8703	0.8285	7.7319	0.8555	0.8489	7.7811	0.9190	0.8846	5.0329	0.6929	0.6917	9.5736
FR	WS-PSNR	0.6985	0.6932	10.294	0.8607	0.8278	7.9919	0.8435	0.8322	8.0719	0.9221	0.8853	4.9415	0.6609	0.6583	9.9652
NR	SSP-BOIQA	0.8600	0.8650	7.3131	0.8772	0.8345	7.6201	0.8532	0.8522	7.5013	0.9054	0.8434	5.4510	0.8544	0.8623	6.8342
NR	MC360IQA	**0.9247**	**0.9187**	**4.6247**	0.9279	0.9190	4.5058	0.9324	0.9252	**4.5825**	0.9344	0.9345	3.7908	0.9220	0.9353	**4.5256**
NR	Zhou et al.	0.8991	0.9232	6.3963	0.9363	**0.9405**	5.6911	0.9200	0.9343	5.8862	**0.9682**	**0.9570**	**3.3304**	**0.9252**	0.9200	4.9721
NR	PW-360IQA	0.9135	0.9155	5.7900	**0.9585**	0.9107	**3.9219**	**0.9398**	0.9107	5.4711	0.9196	0.8929	4.9893	0.9232	**0.9386**	5.3673

**Table 4:** Ablation results on the architecture of the model. The best performance is highlighted in **bold** and the second-best is underlined.

Weights Sharing	VFE	MLP	JND	CVIQ			OIQA			Complexity
				PLCC↑	SRCC↑	RMSE↓	PLCC↑	SRCC↑	RMSE↓	# Params↓
No	✓	✗	✗	0.9261	0.8988	4.4364	0.8819	0.8886	6.6685	58.3 M
	✓	✓	✗	0.9473	0.9409	4.5000	0.8884	0.8913	6.4900	58.4 M
	✓	✗	✓	0.9423	0.9214	4.7900	0.8914	0.8920	6.4100	59.3 M
	✓	✓	✓	0.9372	0.9274	4.9600	0.8989	0.8865	6.2000	59.3 M
Yes	✓	✓	✓	**0.9518**	**0.9507**	**4.4200**	**0.9135**	**0.9155**	**5.7900**	**7.4 M**

## Data Availability

Not applicable.
